# Acute infection with the U.S. isolate of *Theileria orientalis* genotype Ikeda is associated with decreased hematocrit and erythrocyte counts in experimentally infected cattle

**DOI:** 10.3389/fvets.2026.1768408

**Published:** 2026-04-10

**Authors:** Cynthia K. Onzere, Eun-Jee Na, David R. Herndon, Lowell S. Kappmeyer, Chungwon J. Chung, Reginaldo G. Bastos

**Affiliations:** 1 Department of Veterinary Microbiology and Pathology, College of Veterinary Medicine, Washington State University, Pullman, WA, United States; 2 Animal Disease Research Unit, USDA-ARS, Pullman, WA, United States

**Keywords:** cattle, decreased hematocrit, decreased erythrocyte counts, Ikeda U.S. isolate, *Theileria orientalis* Ikeda

## Abstract

**Introduction:**

The emergence of the tick-borne Ikeda genotype of *Theileria orientalis* poses a significant threat to the U.S. cattle industry. Although information has been gathered on parasite distribution, data regarding the pathogenicity of the U.S. isolate of Ikeda remain to be elucidated. In this study, we evaluated the effect of the U.S. Ikeda isolate on cattle experimentally infected with sporozoites, the parasite stage naturally transmitted by ticks.

**Methods:**

Six spleen-intact Holstein calves, aged 6–12 months, were subcutaneously inoculated with 1-mL stabilate containing 3.8 × 10⁵ of *T. orientalis* Ikeda sporozoites. Parasite load, temperature, hematocrit, blood cell counts, and serology were monitored. For this study, we defined acute infection as occurring up to 8 weeks post-inoculation (WPI) and chronic infection as occurring from weeks 10–20. Parasite load was quantified by qPCR targeting the major piroplasm surface protein (MPSP) gene. Hematological parameters were evaluated using a veterinary hematology analyzer. IgM, total IgG, IgG1, and IgG2 profiles were determined by an MPSP-based indirect ELISA.

**Results:**

Parasite load in peripheral blood varied markedly among animals and peaked at 8 WPI (average of 27,078 parasites/μl). A substantial number of parasites were also detected in peripheral blood during the chronic phase (average of 931 parasites/μl). The results demonstrated a significant inverse correlation during the acute phase between parasite load and both hematocrit (*p* = 0.004; *r* = −0.8703) and total erythrocyte count (*p* = 0.001; *r* = −0.9205). However, no significant correlation was found between parasite load and changes in hematocrit or erythrocyte count during the chronic phase. Lymphocyte, neutrophil, and monocyte counts remained within physiological ranges throughout infection. Anti-MPSP IgM was detected at 2 WPI and peaked at 4 WPI, while total IgG appeared at 2 WPI, peaked at 6 WPI, and persisted during the chronic phase. Anti-MPSP IgG1 and IgG2 were detectable from 4 WPI onward, with no significant differences between subclasses.

**Conclusion:**

Acute infection with the U.S. isolate of *T. orientalis* Ikeda was correlated with reduced hematocrit and erythrocyte count in peripheral blood. Infected cattle exhibited robust anti-MPSP antibody responses during the acute phase, which maintained throughout chronic infection.

## 1 Introduction

*Theileria orientalis* is a tick-borne apicomplexan hemoparasite of significant economic importance and represents a serious threat to the bovine industry worldwide (1–3). At least 11 different *T. orientalis* genotypes have been identified based on variations in the major piroplasm surface protein (MPSP) and 18S ribosomal RNA gene (4–6). These genotypes exhibit distinct phenotypes with varying levels of virulence, with Ikeda genotype regarded as the most virulent. Infection with Ikeda is generally associated with anemia, late-term abortion, and mortality; however, asymptomatic cases are not uncommon (1). The global distribution of the Ikeda genotype mirrors that of its primary vector, the Asian longhorned tick (*Haemaphysalis longicornis*). The Ikeda genotype is currently endemic in Asia, South Asia, Australia, and New Zealand (1, 3, 7, 8). Infections of *T. orientalis* Ikeda genotype were first reported in the eastern United States in 2018 (9). These outbreaks were preceded by the detection of *H. longicornis* ticks on the East coast in 2017 (10, 11). Subsequently, it was demonstrated that *H. longicornis* ticks circulating in the United States are competent vectors for the U. S. isolate of *T. orientalis* Ikeda (12). As per the United States Centers for Disease Control and Prevention (CDC) and the USDA Animal and Plants Health Inspection Service (APHIS), *H. longicornis* has currently been identified in more than 20 states (13, 14). Mimicking the distribution of its tick vectors, the U.S. isolate of *T. orientalis* Ikeda parasite has now been reported in multiple states, and is emerging as a growing threat to cattle producers (9, 15–17).

In addition to tick transmission, studies have shown that the piroplasm stages of *T. orientalis* can be mechanically transmitted by insect, such as lice and flies, as well as through iatrogenic means (18–21). However, the epidemiological significance of these transmission routes remains to be elucidated. In contrast to the transforming *Theileria* species *Theileria parva* and *Theileria annulata* (22–25), *T. orientalis* pathogenicity results from the replication of piroplasm stages within erythrocytes, leading to lysis of these cells and the onset of anemia, prostration, anorexia, and even death (1). In this regard, the pathogenicity of *T. orientalis* Ikeda is similar to *Theileria equi* and *Theileria haneyi*, which cause anemia in horses without induction uncontrolled leukocyte proliferation (26–29).

*Theileria orientalis*, like other *Theileria* parasites, has a complex lifecycle that involves the development of distinct parasite stages during its progression through both ticks and cattle (1). *T. orientalis* is transmitted transstadially by ticks (12); however, to date, there is no evidence for transovarial transmission. For the transstadial transmission, larval and/or nymphal ticks ingest the piroplasm (merozoite) stages of the parasite when feeding on infected cattle. Within the tick midgut, piroplasms develop into male and female gametes, which then fuse to form zygotes. Zygotes develop into kinetes that move via the tick hemolymph to reach the salivary glands. Once sporozoites develop in the tick salivary glands, they are transmitted to the host through the tick’s saliva during feeding. Within bovine blood, sporozoites infect leukocytes to form schizonts, which undergo a rapid asexual replication cycle. Schizonts develop into merozoites, which replicate within erythrocytes, leading to anemia, a characteristic of acute infection. Interestingly, iatrogenic or experimental inoculation of naïve animals with blood containing merozoites results in clinical signs of acute infection (18–21). Collectively, this highlights the complexity of the parasite’s life cycle and underscores the need for rigorous studies on the pathogenicity and immunological factors involved in *T. orientalis* infection in cattle. Such studies should utilize sporozoites, which are naturally transmitted by ticks, to fully replicate the parasite’s developmental stages in bovine blood.

While bovines in regions endemic for *T. orientalis* Ikeda genotype develop a resistance to clinical disease following initial infection, naïve animals, particularly newborns and immunocompromised animals, as well as pregnant and lactating heifers and cows, are at risk of developing severe anemia (30, 31). Animals that survive acute disease become lifelong carriers and reservoirs for parasite transmission by ticks and mechanical means (1, 2). Chronically infected cattle typically show no obvious clinical signs; however, stress related to animal management can trigger high parasitemia and anemia, which may negatively affect production (2).

Several knowledge gaps remain regarding the molecular mechanisms underlying the lifecycle of *T. orientalis* as well as the process leading to infection and clinical disease in cattle. Specifically, the impact of acute and chronic infection caused by the U.S. isolate of *T. orientalis* Ikeda on cattle is not fully understood. To address this gap, we experimentally infected cattle with sporozoites, the parasite stage transmitted by ticks in nature, and investigated the level of parasite load during acute and chronic infections and its effect on blood cells, including leukocytes and erythrocytes. Additionally, we examined the profile of immunoglobulins against the immunodominant *T. orientalis* antigen MPSP throughout the infection. This study provided novel and important insights into the progression of infection caused by the U.S. isolate of *T. orientalis* Ikeda, which may help clarify the potential impact of this emerging tick-borne parasite on cattle in the United States.

## 2 Materials and methods

### 2.1 Cattle and parasite

Six spleen-intact healthy Holstein calves, 6 to 12 months of age, were used in this study. The animals were housed in a single pen, kept in a tick-free environment, and monitored daily during the study period. All animal study procedures described in this investigation were approved by the Washington State University Institutional Animal Care and Use Committee (IACUC# 6981) and University of Idaho Institutional Animal Care and Use Committee (IACUC# IACUC 2024–27). Calves were inoculated with salivary gland (SG) stabilate produced from *H. longicornis* infected with the U. S. isolate of *T. orientalis* Ikeda. Production of the SG stabilate was performed following a similar protocol previously described for *T. parva* (32). Briefly, *H. longicornis* nymphs fed to repletion on a *T. orientalis* Ikeda-infected calf for parasite acquisition. Offspring adult ticks were then fed on a naïve calf for 3 days to stimulate sporogony in their salivary glands. The ticks were forcedly removed, and their SG were dissected and suspended at 10 SG/mL stabilate in sterile 7.5% glycerol in complete Roswell Park Memorial Institute 1,640 medium (10% FBS, 20 mM HEPES buffer, 50 μM *β*-mercaptoethanol, 2 mM L-glutamine and 50 μg/mL gentamicin). Parasite load in the SG stabilates was assessed by qPCR, as described below, and determined to be 3.8 × 10^5^ parasites per mL. Animals were inoculated subcutaneously with the SG stabilate (1 mL inoculum) and monitored for parasite load and alterations in temperature, hematocrit, number of leukocytes and erythrocytes in blood, and development of humoral immune response. All six calves were evaluated during the acute phase of the infection while three animals were monitored throughout chronic infection. For the purpose of the study, we define acute infection as occurring within the first 8 weeks post-infection (WPI), and chronic infection as occurring from 10 to 20 WPI. By the end of the study period, the experimental animals were humanely euthanized according to the approved animal care protocols.

### 2.2 Parasite load in peripheral blood

Detection and quantification of *T. orientalis* Ikeda DNA in peripheral blood of infected calves were performed by endpoint PCR and quantitative real-time PCR (qPCR), respectively, targeting the single copy MPSP gene (GenBank accession number: AP011946.1). Briefly, genomic DNA (gDNA) was extracted from 100 uL of EDTA-containing whole blood from the infected animals using the QIAamp DNA Mini Kit (Qiagen), according to the manufacturer’s protocol. For end-point PCR targeting a 776-bp segment of the MPSP gene, the following primers were used: forward 5’ctttgcctaggatacttcct 3′ and reverse 5′ acggcaagtggtgagaact 3′ (12). Reactions consisted of 22.5 uL of AccupPrime™ Pfx Supermix (Thermo Fisher Scientific), 140 nM final concentration of each primer, and 2 uL of DNA template. Amplification was carried out in the C1000 Touch™ Thermal Cycler (Bio-Rad) with an initial denaturation of 95 °C for 5 min and 35 cycles of 95 °C for 15 s, 57 °C for 15 s, and 68 °C for 1 min. Amplicons were visualized via agarose gel electrophoresis. DNA sequencing of PCR products was carried out for specificity confirmation. qPCR was performed on gDNA extracted from whole blood as described above, targeting a 113-bp portion of the MPSP gene from *T. orientalis* Ikeda, as previously reported (33). Briefly, reactions consisted of 1x SsoFast Evagreen™ Supermix (Bio-Rad), 200 nM final concentration of specific primers (forward 5′ ccttcggactacaagcctc 3′ and reverse 5′ tgtgagactcaatgcgccta 3′), and 2 uL of template DNA. The qPCR amplicon was cloned into pCR™4Blunt-TOPO™ (Thermo Fisher Scientific) to construct the control for a standard curve analysis using serial 10-fold dilutions (10^6^ to 10^1^) of plasmid DNA for absolute quantification. qPCR was performed using the CFX Opus Real-Time PCR System (Bio-Rad) with an initial denaturation 98 °C for 2 min followed 40 cycles of 95 °C for 5 s and 58 °C for 5 s. Efficiency of amplification and melt curve analyses were performed to evaluate analytical sensitivity and specificity, respectively, of the MPSP qPCR. Quantification was reported as the number of copies of MPSP per μL of blood.

### 2.3 MPSP indirect ELISA

The presence and level of antibodies against the *T. orientalis* Ikeda MPSP was evaluated by indirect ELISA (iELISA), using recombinant MPSP (recMPSP) as the coating antigen, as previously described (33). Briefly, recMPSP was produced in *Escherichia coli* and purified by nickel columns, using standard protocols. An initial antigenicity evaluation of recMPSP was performed using immunoblot analysis. Briefly, 5 or 10 μg of recMPSP was electrophoresed through 4–20% Mini-PROTEAN^®^ TGX™ Precast Protein Gels (Bio-Rad). After that, protein was transferred into a nylon membrane and incubated with different concentrations of bovine sera from *T. orientalis* Ikeda infected cattle. Membrane was washed and incubated with a secondary anti-bovine IgG labelled with HRP (1:25,000) (Boehringer Ingelheim, Ridgefield, CT, USA) for 1 h. After washing, immune complexes were visualized by enhanced chemiluminescence (ECL substrate, Bio-Rad). recMPSP-based iELISA was performed as previously described (33). Briefly, 96-well Immulon™ 2HB microtiter plates (Thermo Fisher Scientific, Waltham, MA, USA) were coated overnight at 4 °C with 50 μL of recMPSP (2 μg/mL) in 1 × Coating Buffer (BioLegend, San Diego, CA, USA). After that, excess of antigen was removed, and the plates were blocked with 200 μL/well of Blocker™ Casein in PBS (Thermo Fisher Scientific) at room temperature (RT) for 1 h. Following the blocking step, serum samples were diluted 1/50 in 0.05% (v/v) Tween-20 in PBS (PBS-T), and 50 μL was added to duplicate individual wells. Plates were incubated at RT for 1 h, and after four washes in PBS-T, 50 μL of a 1/1000 dilution of anti-bovine IgG peroxidase-labeled secondary antibodies (SeraCare, Milford, MA, USA) were added to individual wells. Plates were then incubated at RT for 1 h. After that, plates were washed four times in PBS-T and developed with 55 μL of 1-Step™ Ultra TMB-ELISA Substrate Solution (Thermo Fisher Scientific). After 10-min incubation in the dark, the enzymatic reaction was stopped by adding 55 μL of TMB Stop Solution (0.2 M H_2_SO_4_) (SeraCare) to each well. Plates were read at optical density (OD) 450 nm using the Synergy HTX ELISA plate reader (BioTek, Winooski, VT, USA). For this study, a preliminary evaluation of the recMPSP-based iELISA was conducted using 131 bovine sera: 28 samples from calves experimentally infected with *T. orientalis* Ikeda and 103 from *T. orientalis*-free calves. iELISA results are presented as ELISA ratio ER, consisting of OD450 test serum / mean OD450 of the negative samples. Test serum samples with an ER ≥ 2 were considered positive.

### 2.4 Blood cell count and hematocrit analysis

Blood cell count and hematocrit were evaluated using the ProCyte One™ Hematology Analyzer (IDEXX Laboratories, Inc., Westbrook, ME), following the manufacturer’s protocol. Peripheral blood was collected into Vacutainer^®^ tubes containing EDTA (BD Company, Franklin Lakes, NJ) at several timepoints post-inoculation. After collecting, whole blood samples were homogenized for 5 min, and the numbers of total white blood cells (WBC), lymphocytes, monocytes, neutrophils, and erythrocytes were measured. Results of white blood cell counts were presented as 1,000 cells/mL of blood, and erythrocytes counts were presented as 1,000,000 cells/mL of blood. Hematocrit was assessed as the percentage of erythrocytes relative to the total blood volume.

### 2.5 Statistical analysis

Association between parasite load in peripheral blood and changes in hematocrit or erythrocyte counts were assessed using Pearson’s r correlation in GraphPad Prism 10.3.1 (GraphPad Software, San Diego, CA). A significance threshold of 0.05 was used to determine statistical significance in the correlation analyses.

## 3 Results

### 3.1 Parasite load in peripheral blood following sporozoite inoculation of cattle

**Figure 1:**
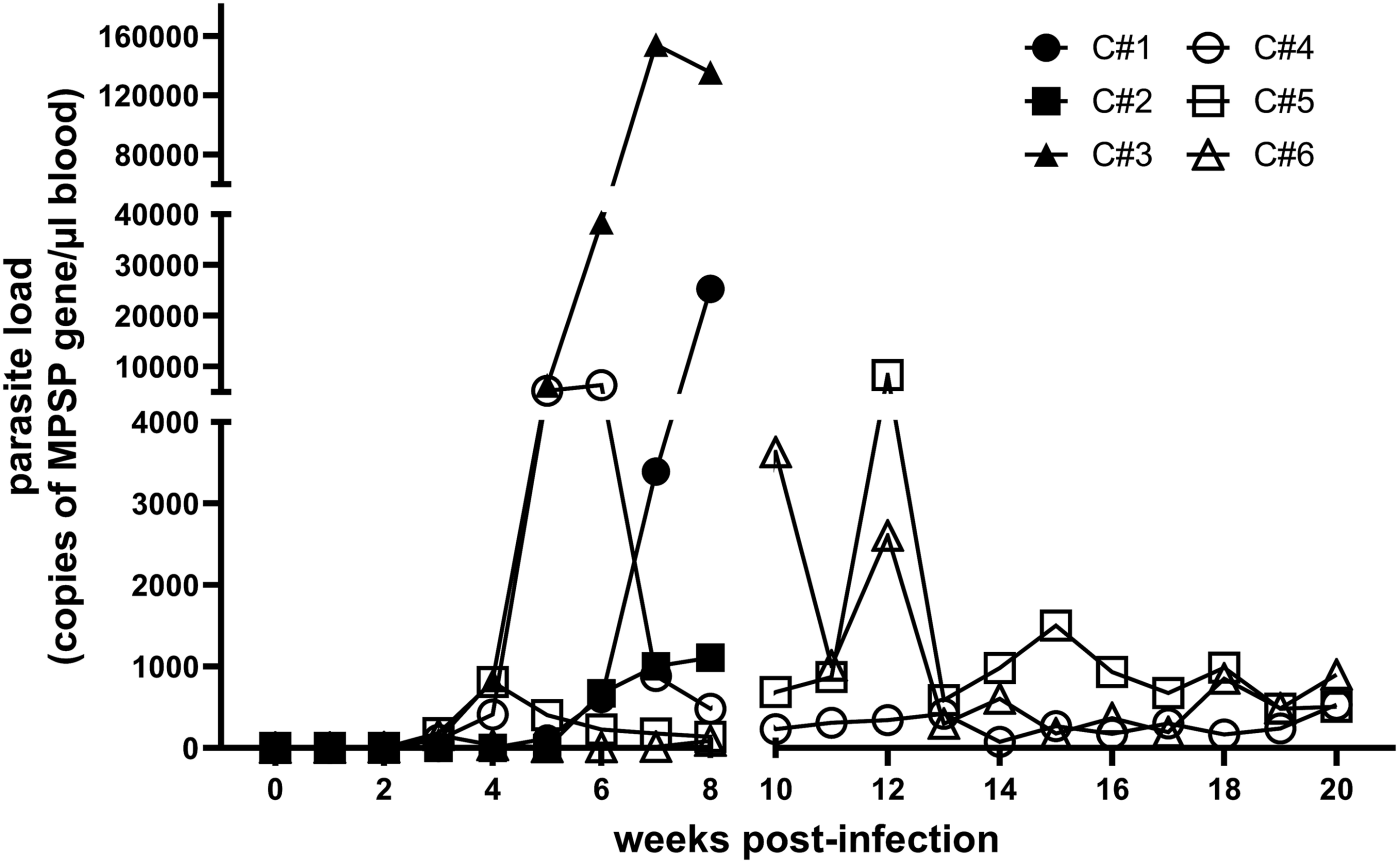
*Theileria orientalis* Ikeda levels in peripheral blood of cattle following inoculation of parasite sporozoites. Results are presented as copies of the piroplasm surface protein (MPSP) gene per μL of blood. Lines with black symbols represent animals evaluated during the acute phase of the infection only while lines with open symbols indicate animals evaluated during both the acute and chronic phases of infection.

The presence of *T. orientalis* DNA was detected by endpoint PCR, starting at 3–6 WPI and remained consistently detectable until the end of the experiment at 20 WPI (Supplementary Table S1). qPCR results demonstrated a large variation in parasite load in peripheral blood among the experimental calves. The parasite load of animals #1–4 peaked between weeks 6 to 8 post-inoculation. In contrast, the parasite load of calves #5 and #6 peaked at 12 WPI and 10 WPI, respectively (Figure 1). The three calves evaluated throughout the chronic phase of the infection (C#4–6) maintained significant levels of parasite in peripheral blood (average 645 (±220) parasites/μl of blood), demonstrating substantial parasitaemia during chronicity (Figure 1). Interestingly, despite differences in parasite load, the infected animals exhibited a similar pattern of parasite burden, characterized by a peak usually during the acute phase of infection, followed by a stable level of parasites in the peripheral blood that persisted throughout the chronic phase. In summary, the data demonstrated that calves inoculated with sporozoites of the U.S. *T. orientalis* Ikeda developed significant levels of parasitemia in peripheral blood during the first peak of parasite replication, which occurred mainly between 6 to 8 WPI. Additionally, the results indicate that chronically infected animals maintain detectable, elevated levels of circulating parasites in peripheral blood.

### 3.2 Acute infection with the U.S. isolate of *T. orientalis* genotype Ikeda significantly reduces hematocrit and erythrocyte counts in cattle

**Figure 2:**
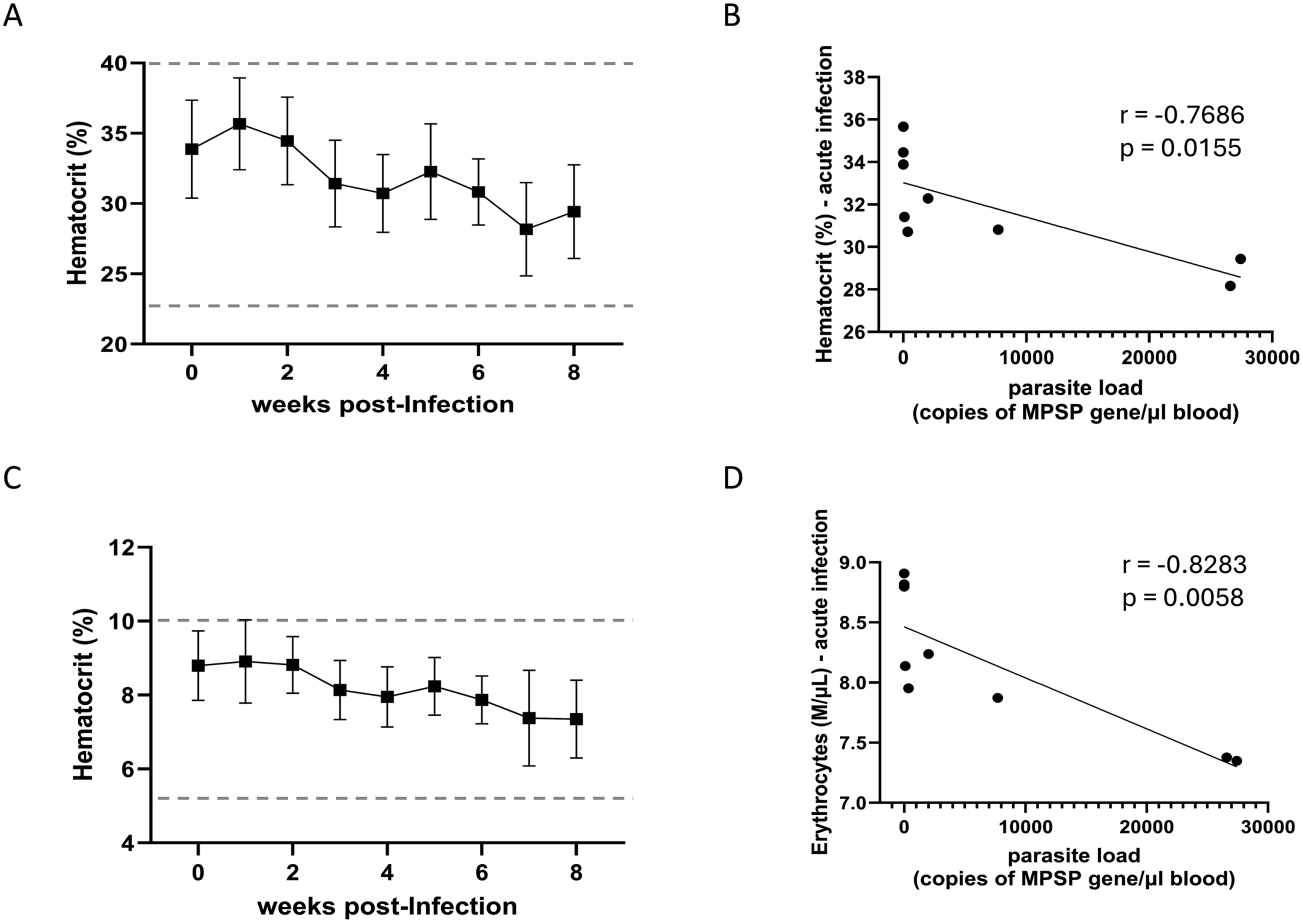
Hematocrit **(A)** and correlation between hematocrit and parasite load **(B)** in cattle acutely infected with the U.S. isolate of *Theileria orientalis* Ikeda. Number of erythrocytes in peripheral blood **(C)** and correlation between erythrocyte counts and parasite load **(D)** in cattle acutely infected with the U.S. isolate of *T. orientalis* Ikeda. Results of hematocrit are presented as percentage while erythrocytes are shown as 1,000 cells per μL of peripheral blood. Parasite load in peripheral blood is expressed as copy number of the major piroplasm surface protein (MPSP) gene per μL of blood. The *r* values indicate the Spearman *r* correlation coefficient.

**Figure 3:**
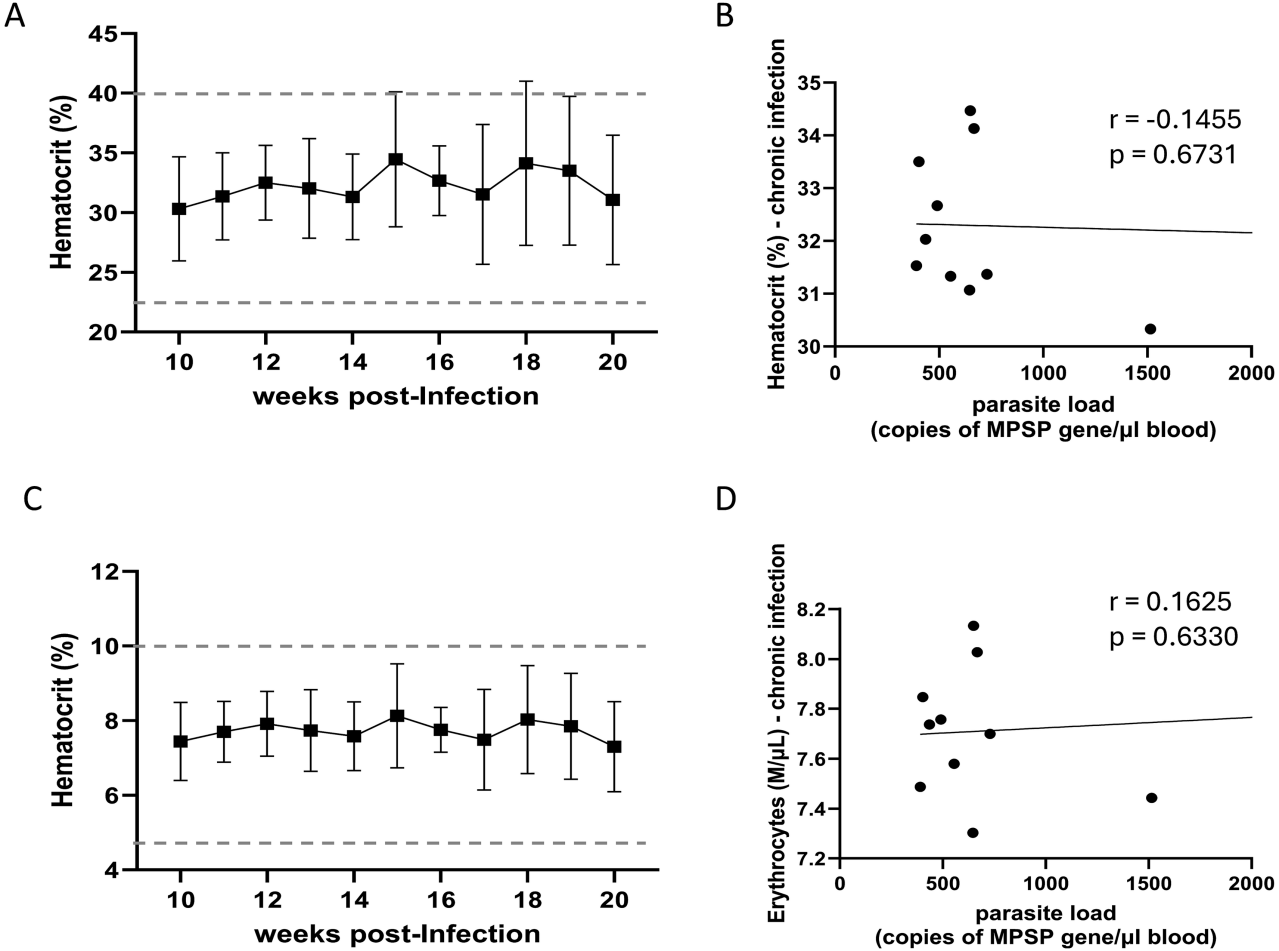
Hematocrit **(A)** and correlation between hematocrit and parasite load **(B)** in cattle chronically infected with the U.S. isolate of *Theileria orientalis* Ikeda. Number of erythrocytes in peripheral blood **(C)** and correlation between erythrocyte counts and parasite load **(D)** in cattle chronically infected with the U.S. isolate of *T. orientalis* Ikeda. Results of hematocrit are presented as percentage while erythrocytes are shown as 1,000 cells per μL of blood. Parasite load in peripheral blood is expressed as copy number of the major piroplasm surface protein (MPSP) gene per μL of blood. The *r values indicate the Spear*man *r* correlation coefficient.

To investigate the effect of the U.S. isolate of *T. orientalis* Ikeda on experimentally infected cattle, we monitored temperature, hematocrit, and total erythrocyte counts in peripheral blood during both the acute and chronic phases of infection (Figures 2, 3 and Supplementary Tables S2–S4). No fever was observed in the infected animals throughout the study period (Supplementary Table S4). Although hematocrit values remained within the normal range for cattle during the acute phase of infection, a significant inverse correlation was observed between parasite load in peripheral blood and hematocrit levels (*r* = −0.8703; *p* = 0.004; 95% confidence interval) (Figures 2A,B). Similarly, while the total erythrocyte counts in peripheral blood stayed within the physiological range during acute infection, a significant inverse correlation was found between parasite load and erythrocyte count (*r* = −0.9205; *p* = 0.001; 95% confidence interval) (Figures 2C,D). During the chronic phase, both hematocrit values and total erythrocyte counts remained within normal ranges, and no significant correlations were observed between parasite load and changes in these parameters in calves chronically infected with the U.S. isolate of *T. orientalis* Ikeda (Figure 3). Figures 2, 3 show the effects of the U.S. isolate of *T. orientalis* Ikeda on hematocrit and erythrocyte counts during the acute and chronic phases of infection in cattle, respectively.

### 3.3 Blood leukocytes profile during acute and chronic infections

**Figure 4:**
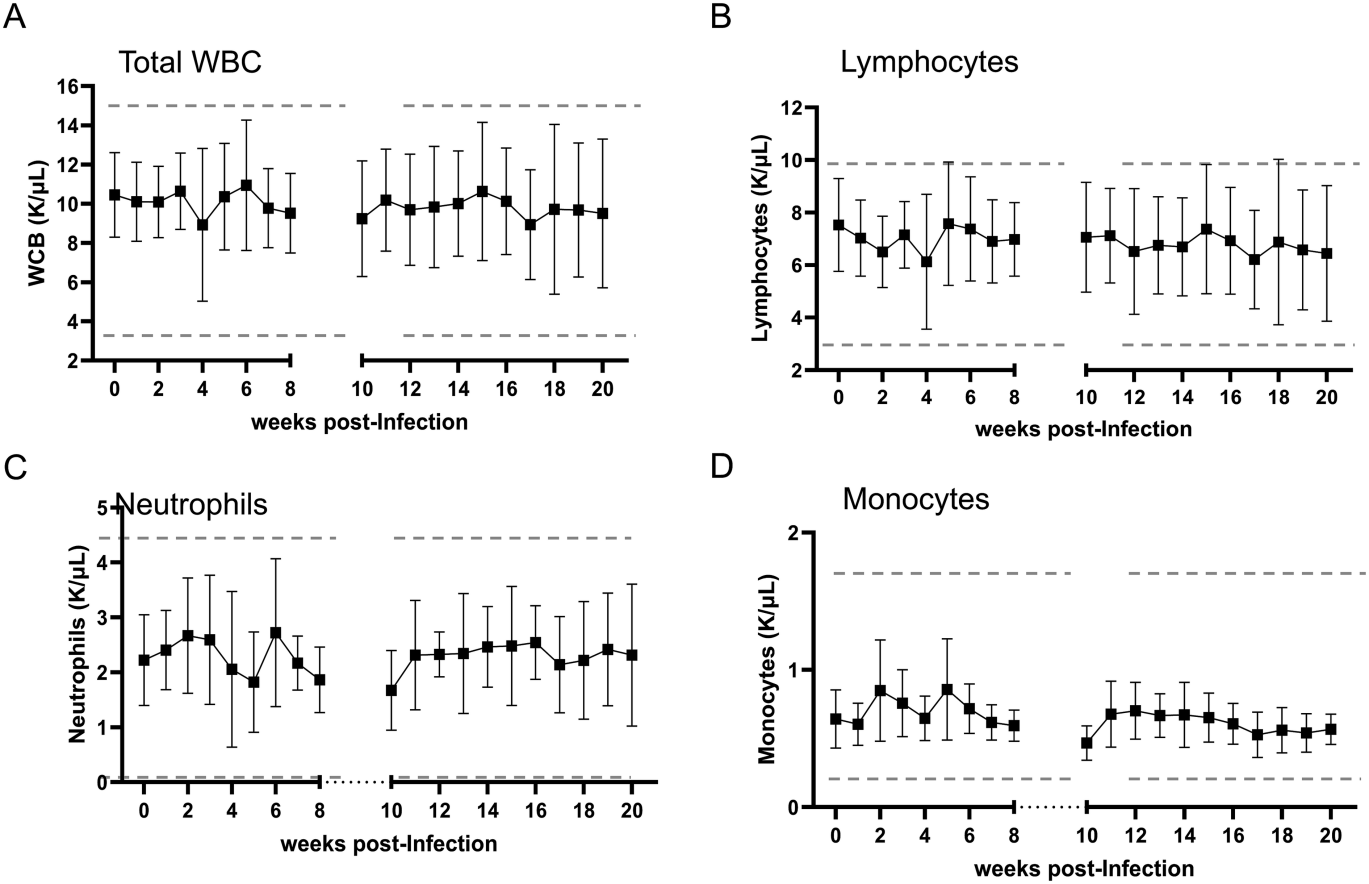
Absolute numbers of bovine total white blood cells (WBC) **(A)**, lymphocytes **(B)**, neutrophils **(C)**, and monocytes **(D)** in peripheral blood following infection with the U.S. isolate of *Theileria orientalis* Ikeda sporozoites. Upper and lower dashed lines represent the maximum and minimum physiological cell values, respectively.

Total numbers of WBC, lymphocytes, monocytes, and neutrophils in peripheral blood were monitored throughout the acute and chronic phases of infection (Figure 4). Although two infected calves exhibited a marginal decrease in WBCs at 4 WPI and another showed an increase at 6 WPI, the overall WBC counts remained within the normal range throughout the study period (Figure 4A). One calf displayed elevated lymphocyte levels between weeks 5 and 6 post-infection; however, no significant changes were observed in the other infected animals (Figure 4B). Mild fluctuations in neutrophil and monocyte counts were noted during weeks 3 and 6, and weeks 2 and 5 post-inoculation, respectively; however, these variations were not statistically significant during either phase of infection (Figures 4C,D). In summary, no significant alterations in total WBC, lymphocytes, neutrophils, and monocytes were observed in calves infected with the U.S. isolate of *T. orientalis* Ikeda throughout the study period.

### 3.4 Humoral immune response during acute and chronic infections

**Figure 5:**
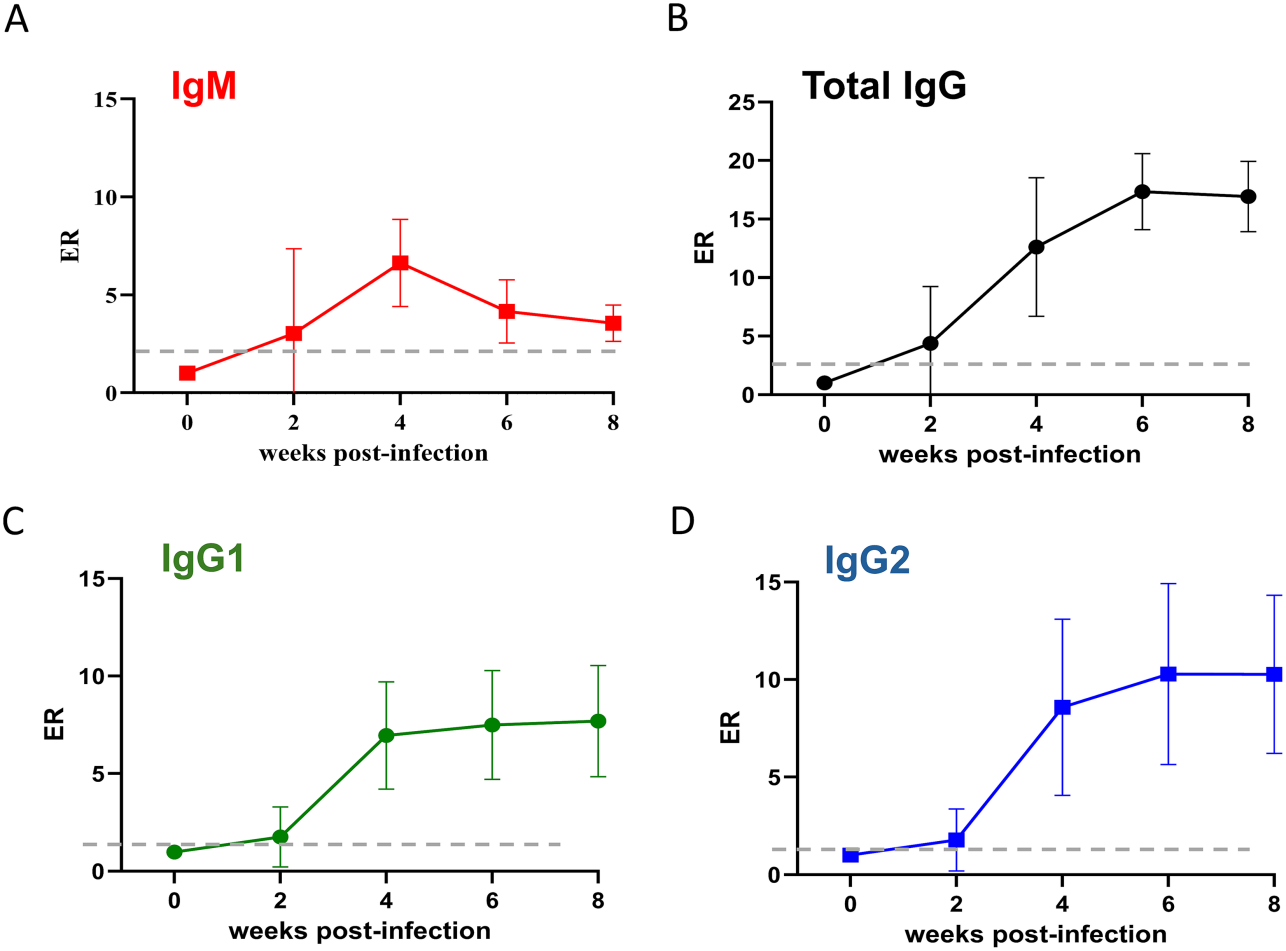
Levels of anti-major piroplasm surface protein IgM **(A)**, total IgG **(B)**, IgG1 **(C)**, and IgG2 **(D)** of cattle (*n* = 6) during the acute phase of infection with the U.S. isolate of *Theileria orientalis* Ikeda. Results are presented as an ELISA ratio (ER) (ER = mean OD450 test serum/mean OD450 of negative control sera). Dashed lines indicate the cutoff; serum samples with an ER ≥ 2 were considered positive.

**Figure 6:**
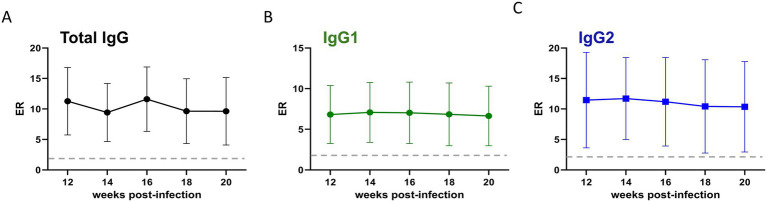
Levels of anti-major piroplasm surface protein total IgG **(A)**, IgG1 **(B)**, and IgG2 **(C)** of cattle (*n* = 3) during the chronic phase of infection with the U.S. isolate of *Theileria orientalis* Ikeda. Results are presented as an ELISA ratio (ER) (ER = mean OD450 test serum/mean OD450 of negative control sera). Dashed lines indicate the cutoff; serum samples with an ER ≥ 2 were considered positive.

Given the current knowledge gap regarding the humoral response following the infection with the U.S. isolate of *T. orientalis* Ikeda, we investigated the levels of IgM, total IgG, IgG1, and IgG2 using an MPSP-based iELISA (Supplementary Figures S1 and Figures 5, 6). As an initial step in developing the iELISA, the immunogenicity of recMPSP was investigated using bovine sera from *T. orientalis*-infected animals from previous studies conducted in our laboratories (12, 33, 34) (Supplementary Figure S1). Immunoblot analysis demonstrated that sera from infected animals recognized a single protein band of 32KDa, corresponding to the expected size of recMPSP. Next, we conducted an experimental validation of the recMPSP-based iELISA using 28 samples from calves experimentally infected with *T. orientalis* Ikeda and 103 samples from *T. orientalis*-free animals available in our laboratories (Supplementary Figure S1B). Subsequently, the recMPSP ELISA was used to investigate the profile of IgM, total IgG, IgG1 and IgG2 during acute and chronic infection.

The results demonstrate that the animals developed a canonical pattern of anti-MSPS IgM and total IgG responses during acute infection. Detectable levels of IgM were observed at 2 WPI, peaking at 4 WPI (Figure 5A). Similarly, total IgG levels became detectable at 2 WPI, peaked at 6 WPI, and remained detectable throughout the study period (Figure 5B). The pattern of both IgG1 and IgG2 mirrored that of total IgG and no significant differences were observed in the ratio between these two IgG subclasses throughout the acute phase of the infection (Figures 5C,D). Next, we investigated the levels of total IgG, IgG1, and IgG2 in calves chronically infected with the U.S. isolate of *T. orientalis* Ikeda (Figures 6A–C). The results demonstrated that the animals maintained detectable and consistent levels of anti-MPSP IgG, comprising total IgG, IgG1, and IgG2, throughout chronic infection.

## 4 Discussion

The recent emergence of the *T. orientalis* Ikeda genotype in the United States poses a significant risk to the nation’s cattle industry (9, 15, 17, 35). While the origin of the Ikeda isolate currently circulating in the United States remains unclear, its emergence may be attributed to the importation of infected animals and/or animals infested with *H. longicornis* carrying the parasite (9). Following its introduction in 2018, a few studies have been conducted on the prevalence of *T. orientalis* Ikeda in the United States (9, 15, 16, 35). However, given the recent appearance of this genotype in the country, little is currently known about its pathogenicity. To begin addressing this gap, in this study, cattle were infected with the U. S. isolate of *T. orientalis* Ikeda and monitored during both acute and chronic phases of infection for alterations in clinical parameters, blood cell counts, as well as for their humoral immune response against the well-characterized immunodominant antigen MPSP. To most closely mimic the natural transmission of the disease, the animals in this study were infected with sporozoites, the parasite stage that is naturally transmitted by ticks. The results indicate that acute infection with the U. S. isolate is associated with a significant reduction in hematocrit percentage and a decrease in erythrocyte counts in peripheral blood. Infected animals developed substantial levels of anti-MPSP antibodies, which maintained throughout the chronic phase of infection.

*Theileria* infection is typically characterized by markedly fluctuating parasitemia, which peaks during the acute phase of the disease and remains at low but detectable levels during the chronic phase (28, 36). We confirmed this observation by demonstrating that calves infected with sporozoite stabilates (3.8 × 10^5^ parasites/mL) exhibited substantial variation in parasite load in peripheral blood. The number of parasites in the SG stabilate was measured by qPCR therefore, it reflected sporozoite quantity rather than viability. Despite this intrinsic restraint of the present study, the variation in parasite load among infected animals, particularly during acute infection, suggests that factors beyond infective dose and parasite viability may influence parasite load. Cattle genetic factors, such as the major histocompatibility complex (MHC) and breed have been shown to influence susceptibility to *T. orientalis* Ikeda (37). Investigating the MHC haplotypes of infected animals and potential bovine genetic factors influencing infection was beyond the scope of this study. Future studies are needed to determine whether the development of high loads of *T. orientalis* Ikeda and the resulting severe clinical symptoms with specific MHC haplotypes or other genetic factors.

Although the taxonomy of *T. orientalis* genotypes remains a subject of ongoing debate (4, 38), the Ikeda genotype is widely recognized for its association with clinical disease, typically manifesting as anemia, late-term abortions, and even death (1, 2). Nevertheless, asymptomatic infections with this genotype are also commonly reported. The factors contributing to the variation in clinical presentation of infections with the *T. orientalis* genotypes, especially for the Ikeda genotype, are currently unknown. From an epidemiological perspective, it is worth noting that asymptomatic animals infected with Ikeda genotype act as reservoirs for the parasite, enabling both tick-borne and iatrogenic transmissions (1, 2, 20). The threat posed by the recent introduction of the Ikeda genotype into the United States is further exacerbated by the uncontrolled spread of *H. longicornis* ticks, the primary vector of *T. orientalis* Ikeda, across the country (39–43). The pathogenesis of *T. orientalis* Ikeda remains poorly understood, particularly for the U.S. isolate, for which data are scarce. Similarly, the potential modulatory effect of the parasite’s schizont stage on leukocytes in infected cattle remains unclear. In this study, calves were infected with sporozoites, the parasite stage naturally transmitted by ticks, to closely reproduce the natural infection of Ikeda in the field (1, 2). The results indicated that infection did not affect peripheral blood leukocyte counts, consistent with a previous report (44). This finding also demonstrates that *T. orientalis* Ikeda infection differs from infections caused by related *Theileria* and *Babesia* species, which are associated with marked changes in peripheral blood leukocytes (28, 45–47).

*Theileria orientalis* Ikeda has been considered an economically important parasite of cattle in endemic countries such as Japan, Australia and New Zealand (1–3). Studies conducted in these endemic areas indicate that the clinical presentation of disease varies but is particularly severe in naïve animals introduced into infected herds, newborns, and those under stress (2). Effects of the Ikeda infection on milk production and weigh gain remain controversial. While some studies report a significant reduction in production (48), others suggest that the parasite has little or no effect on cattle in endemic herds (49). Given that *T. orientalis* Ikeda has only recently emerged in the United States, its impact on cattle during both acute and chronic infections remains largely unknown, aside from a few recent studies documenting its prevalence and sporadic clinical cases (15, 35). Taken together, the results of this present study provide novel insights into the pathogenesis of the U.S. isolate of *T. orientalis* Ikeda. It is important to note that our results were obtained from experimentally infected animals with minimal management stress. Consequently, production animals are expected to exhibit more severe clinical signs, as observed in endemic regions with Ikeda genotype isolates in Japan, New Zealand, and Australia, and more recently in the United States (2, 3, 9, 44). Additionally, this study focused exclusively on hematocrit and erythrocyte counts; no additional parameters, such as clinical scoring or markers of hemolysis, were evaluated. Logistic constraints also precluded the use of a large sample size in this cattle study. Altogether, these limitations should be considered when extrapolating these findings to production animals.

It has been demonstrated that MPSP is expressed in both sporozoite and merozoite stages of *T. orientalis* (50). Furthermore, the immunodominant nature of MPSP has been previously reported (33, 51). Collectively, these findings support the use of MPSP as a suitable antigen for serological assays aimed at detecting cattle exposed to *T. orientalis*. In this study, we produced a recombinant version of MPSP from the U.S. isolate of *T. orientalis* Ikeda, which was recognized by antibodies from experimentally infected animals. These results provided the foundation for developing an iELISA employed in this study to investigate the profile of immunoglobulins during acute and chronic infection of the U.S. isolate of *T. orientalis* Ikeda in experimentally infected cattle. To the best of our knowledge, the profile of anti-MPSP immunoglobulin isotypes and subclasses following *T. orientalis* Ikeda infection has not been previously described. Therefore, our study is the first to characterize this profile, showing that IgM against MPSP peaks concurrently with the beginning of parasitemia in peripheral blood around week 4 post-infection. The levels of total IgG against MPSP described in this study peaked at week 6 post-infection, corroborating previously published data (51). To provide further insight into the elicitation of anti-MPSP IgG during *T. orientalis* Ikeda infection, we demonstrated that infected animals maintain significant levels of this immunoglobulin up to 20 WPI, a period regarded as the chronic phase of infection. The bovine IgG class contains three subclasses, IgG1, IgG2, and IgG3 (52, 53), with the first two being the most studied due to the availability of reagents, such as specific HRP conjugates. It has been demonstrated that bovine IgG subclasses exhibit distinct effector functions mediated by their Fc regions, and these functional differences can influence the outcome of infection. In addition, protection of cattle against *Anaplasma marginale* was associated with IgG2 titers, which exhibit optimal opsonizing activity (52, 54). Here we report significant levels of anti-MPSP IgG1 and IgG2 responses during both acute and chronic infection with the U.S. isolate of *T. orientalis* Ikeda, but no significant differences were observed between the levels of the IgG subclasses. Collectively, it can be speculated that anti-MPSP IgG may contribute to reducing parasitemia during the acute phase; however, they are insufficient to eliminate the parasite or preventing the establishment of chronic infection. Further studies are needed to investigate the impact of Ikeda infection on bovine cellular and humoral immune mechanisms, which may have implications for clinical disease progression, vaccine development, and the establishment of chronic infection.

In this study, animals were inoculated with parasite stabilate prepared from SG of *H. longicornis* infected with the U.S. isolate of the Ikeda genotype. The rationale behind this strategy was to closely mimic natural infection (1, 2, 12). However, this approach has some caveats, as not all components of tick saliva are fully represented in the SG stabilate. Further investigations involving a larger number of animals and tick transmission are necessary to better understand the interaction between *H. longicornis* and the U.S. isolate of *T. orientalis* Ikeda, and how this relationship influences parasite pathogenesis, host immune responses, and the establishment of parasite persistence. The *T. orientalis* Ikeda genotype used in this study originated from the first isolate identified in the United States in 2018 (9) and has been used in previous investigations (12, 33, 34). However, given that this genotype has been circulating in the country for more than 6 years, it is plausible that genetically diverse populations are now present. While this does not compromise the impact of the present study, it underscores the importance of maintaining robust surveillance programs for cattle populations in the United States and other endemic regions, as well as further research on the pathogenesis of *T. orientalis* Ikeda.

## 5 Conclusion

We conclude that acute infection with the U.S. isolate of *T. orientalis* Ikeda genotype is significantly associated with reduced hematocrit and erythrocyte counts in peripheral blood of cattle. These findings were obtained under experimental conditions; therefore, it is plausible that the pathogenicity of the U.S. isolate may be even more pronounced in production animals exposed to greater management-related stress. The results demonstrate a significant immunoglobulin response to the immunodominant MPSP antigen following infection with *T. orientalis* Ikeda, which is maintained throughout the chronic phase of infection. Collectively, these findings provide important insights into the pathogenic potential of this emerging parasite and underscore its threat to the U.S. cattle industry. Although conducted under controlled conditions, this study offers valuable information on pathogenicity factors of the U.S. isolate of *T. orientalis* Ikeda and lays the groundwork for future research on its impact on cattle productivity.

## Data Availability

The original contributions presented in the study are included in the article/Supplementary material, further inquiries can be directed to the corresponding author.
