# Supplementary figures and images for: Acute infection with the U.S. isolate of Theileria orientalis genotype Ikeda is associated with decreased hematocrit and erythrocyte counts in experimentally infected cattle
Source: Front Vet Sci. 2026 Apr 10;13:1768408. doi: 10.3389/fvets.2026.1768408 (PMC13106022; doi:10.3389/fvets.2026.1768408)

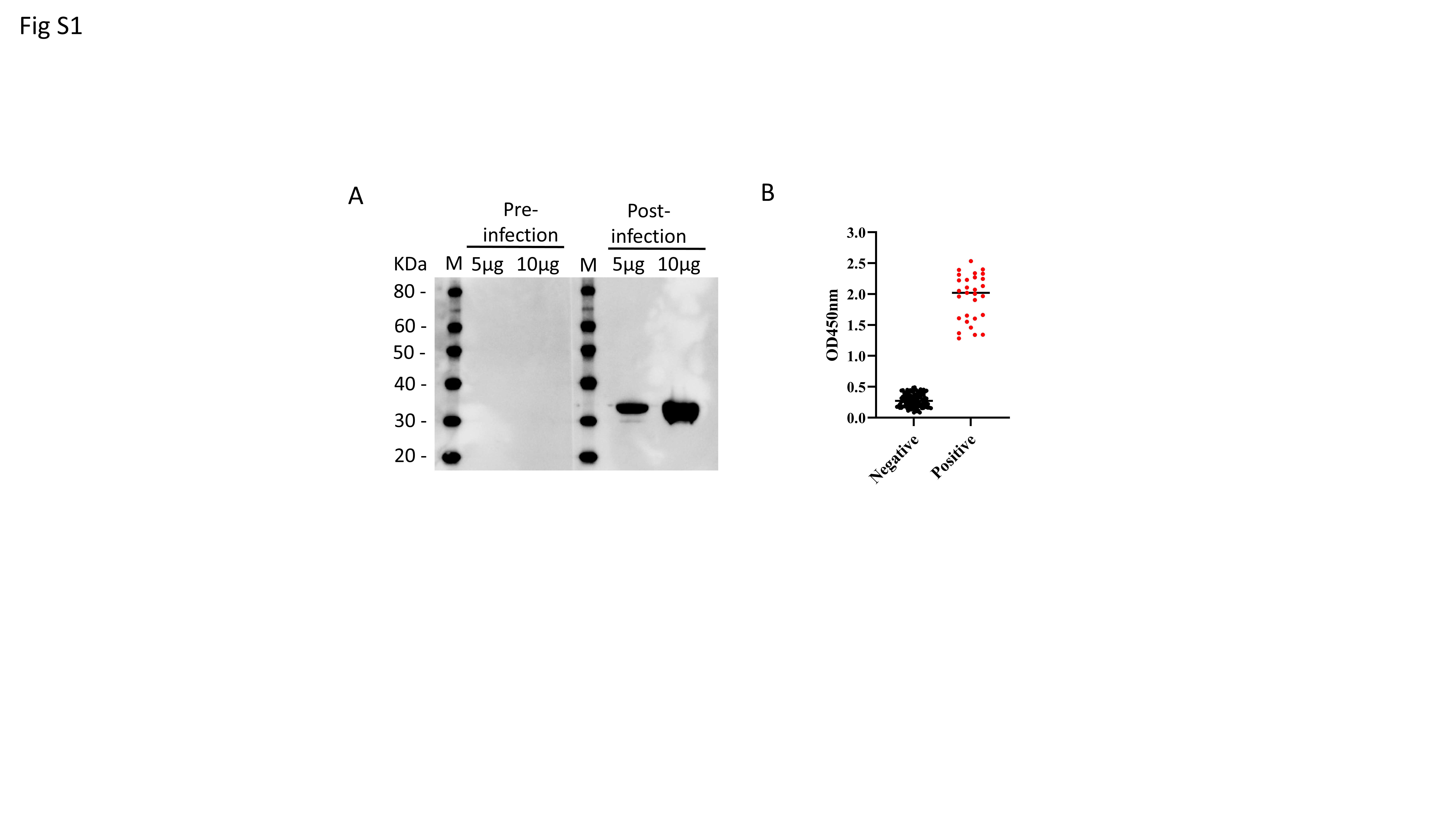

Supplement: Supplementary file 1 [file Image_1.tif]
